# Supplementary material for: Complexome profiling of the Chlamydomonas psb28 mutant reveals TEF5 as an early PSII assembly factor
Source: Plant Cell. 2025 Mar 17;37(6):koaf055. doi: 10.1093/plcell/koaf055 (PMC12164590; doi:10.1093/plcell/koaf055)
Supplement: koaf055_Supplementary_Data [file koaf055_supplementary_data.zip › psb28_interactive.zip!/plots/intensity/qualityPlots/peptides/PRPS1_Cre09.g394750.t1.2.html]

### Cre09.g394750.t1.2

Trivial name: PRPS1  
SeqCoverage [median]: 5.30  
Mass: 48.30 kDa  
  
 

| Sequence | MissedCleavage | Mass [Da] | wtIntensity | muIntensity | Score |
| --- | --- | --- | --- | --- | --- |
| ASSTVSNDIQGFK | 0 | 1352.66 | 0.00 | 394.37 | 93.26 |
| FLEVDEEAER | 0 | 1235.57 | 0.00 | 1153.35 | 81.59 |
|  |  |  | 0.0 | 1547.7 |  |
